# Real-time monitoring of liver fibrosis through embedded sensors in a microphysiological system

**DOI:** 10.1186/s40580-021-00253-y

**Published:** 2021-02-02

**Authors:** Hafiz Muhammad Umer Farooqi, Bohye Kang, Muhammad Asad Ullah Khalid, Abdul Rahim Chethikkattuveli Salih, Kinam Hyun, Sung Hyuk Park, Dongeun Huh, Kyung Hyun Choi

**Affiliations:** 1 Department of Mechatronics Engineering, Jeju National University, Jeju-si, Republic of Korea; 2 Department of Bioengineering, University of Pennsylvania, Philadelphia, USA

**Keywords:** Liver fibrosis-on-chip, TEER sensor, ROS sensor, Embedded sensors, TGF-β1

## Abstract

Hepatic fibrosis is a foreshadowing of future adverse events like liver cirrhosis, liver failure, and cancer. Hepatic stellate cell activation is the main event of liver fibrosis, which results in excessive extracellular matrix deposition and hepatic parenchyma's disintegration. Several biochemical and molecular assays have been introduced for in vitro study of the hepatic fibrosis progression. However, they do not forecast real-time events happening to the in vitro models. Trans-epithelial electrical resistance (TEER) is used in cell culture science to measure cell monolayer barrier integrity. Herein, we explored TEER measurement's utility for monitoring fibrosis development in a dynamic cell culture microphysiological system. Immortal HepG2 cells and fibroblasts were co-cultured, and transforming growth factor β1 (TGF-β1) was used as a fibrosis stimulus to create a liver fibrosis-on-chip model. A glass chip-based embedded TEER and reactive oxygen species (ROS) sensors were employed to gauge the effect of TGF-β1 within the microphysiological system, which promotes a positive feedback response in fibrosis development. Furthermore, albumin, Urea, CYP450 measurements, and immunofluorescent microscopy were performed to correlate the following data with embedded sensors responses. We found that chip embedded electrochemical sensors could be used as a potential substitute for conventional end-point assays for studying fibrosis in microphysiological systems.

## Introduction

Hepatic tissue's regenerative capacity safeguards the liver from acute injury even when a significant part of the liver is damaged or excised [1]. Liver is capable of restoring its histology and physiology during the process of regeneration. However, chronic liver abnormalities result in impaired regenerative capacity, which results in a robust inflammatory response, accelerated apoptosis, necrosis, and eventually scar tissue formation (wound healing process). The wound healing process is instrumental in acute liver injuries; however, in the case of chronic liver pathologies, scar tissue disintegrates the liver parenchyma, interrupt blood flow, and eventually results in disruption of cohesive cell–cell tight junction proteins (TJPs) [2, 3]. The scar tissue is mainly composed of the extracellular matrix (ECM) proteins collagens, proteoglycans, and glycoproteins, especially cross-linked collagen type I and type III [1]. Its abnormal aggregation is called liver fibrosis. Hypoxic injury or oxidative stress and necroinflammation are considered allied mediators of hepatic stellate cell (HSC) activation, the primary liver fibrosis mediator. Other factors that trigger HSC activation include reactive oxygen species (ROS), lipopolysaccharide, paracrine cytokine stimulation, apoptotic bodies, and resident Kupffer cells [4]. The advanced form of liver fibrosis is referred to as cirrhosis. Fibrotic liver and cirrhosis are the main risk factors for liver cancer and have become a significant concern worldwide [5]. Cellular oxidative stress (OS) is a state in which cell antioxidant and pro-oxidant redox balance permute in favor of pro-oxidant condition, which leads to considerable cell damage. Increased production of reactive nitrogen species or ROS or decreased antioxidant levels are the leading causes of OS. ROS perform several vital roles in physiological conditions such as host defense against microbes, cell signaling pathways, and cell cycle regulation [6]. ROS are extremely unstable and consist of hydrogen peroxide (H_2_O_2_), superoxide anions (O_2_^**.−**^), and hydroxyl radicals (HO·) [7]. However, O_2_^**.−**^ is extremely unstable; hence, superoxide dismutase (antioxidant enzyme) promptly converts it into H_2_O_2._ Which is relatively stable, long-lived, and lacks ionic charge, making it freely diffusible and catastrophic to intracellular macromolecules, including cell–cell tight junction proteins. An increased ROS level in the endoplasmic reticulum, especially H_2_O_2_, results from excessive utilization of reduced glutathione ends up in increased oxidized misfolded proteins and release of calcium from ER [8]. It eventually results in oxidative stress in mitochondria, ultimately leading to apoptosis and cellular injury. Unfortunately, no effective anti-fibrosis treatment is available, and current therapies for curing liver fibrosis and cirrhosis are primarily limited to alleviating chronic stresses. The lack of robust and repeatable in vitro models for liver fibrosis is one of the primary obstacles in discovering efficient treatments [9].

In recent years, organ-on-chip (OOC) technology based on microfluidics emerged as a potential replacement for animal modeling. In this technology, microfluidic chips provide an in-vivo microenvironment and nurture biological tissue with physiological shear stress. Hence, these are becoming a popular choice for disease modeling, reverse engineering of human organs, and pharmaceutical testing. OOC is also termed as microphysiological systems (MPS) [10]. To date, several liver-on-chip (LOC) models have been introduced for studying hepatic anomalies [11, 12] and compound toxicity [13, 14]. Lee et al. developed a liver fibrosis-on-a-chip model to investigate the impact of gelatin bioinks through cell printing technology. They used pre-activated HSC; however, liver fibrosis development was not examined in real-time [15]. Jang et al. identified liver fibrosis biomarkers based on the activation of stellate cells and liver function enzymes using a liver-chip [14]. Huang et al. proposed an immunosensor for screening liver fibrosis markers on a chip, but it has not been tested in microfluidics with hepatic cells [16]. Zhou et al. studied paracrine cell signaling within liver-on-a-chip and targeted liver fibrotic biomarker TGF-β1 [17].

Trans-epithelial electrical resistance (TEER) and electrical cell-substrate impedance sensing (ECIS) are the unique approaches to evaluate real-time impedance measurement of biological tissues [18]. TEER estimation is a quick and non-invasive method to measure in vitro cells' differentiation and the epithelial monolayer integrity [19]. It was previously employed in OOC to assess the cell monolayer barrier integrity, cell viability, cell–cell tight junction formation, and drug toxicity response [13, 20, 21]. However, TEER impedance spectroscopy has not been used to monitor liver fibrosis development in a microfluidic system. Commercial TEER and ECIS modules such as Millicell ERS, EVOM2, REAL-TIME Roche xCELLigence Analysis (RTCA), and AutoLab potentiostat are mostly used in the reported literature [20–27]. The micro-size of microphysiological organ chips makes it harder to incorporate the impedance measuring electrodes into the microfluidic systems; however, previously, electrodes have been incorporated into microfluidic chips made of polydimethylsiloxane (PDMS) [21]. We explored this opportunity and used a validated embedded indium tin oxide (ITO) based transparent TEER sensor for studying liver fibrosis.

Fluorescent microscopy and chemiluminescence assays are a popular choice among researchers for ROS measurement [28–30]. However, a few efforts have been made for the electrochemical detection of ROS in microfluidic systems. Ko et al. employed bimetallic nanoparticles to continuously detect hydrogen peroxide in microfluidic devices for up to 100 min [31]. Jin et al. modified a microfluidic chip by integrating a flexible electrochemical sensor for monitoring vascular transduction and evaluated the effect of H_2_O_2_ on vascular mechanotransduction [32]. End-point assays are typical for the evaluation of LOC models, but biologicals processes occur in real-time. Hence, end-point assessment cannot picture the actual biological events happening in a MPS [33]. In this study, considering ECM, TJPs, and ROS's role, a liver fibrosis-on-chip model was modeled to check the feasibility of integrated TEER and newly developed ROS sensors for monitoring fibrosis development. TEER sensor was printed on-chip using chemical vapor deposition (CVD) technique, which has been previously described [34]. While the ROS sensor pattern used a solution-based inkjet printing technique on the glass chip to detect H_2_O_2_. The effect of different ECM on the attachment of liver and fibroblasts cells to the microfluidic glass chip was also evaluated. The sensor data revealed that the chip embedded TEER sensor and ROS sensor could monitor liver fibrosis development. We further performed biomarker assays to correlate the integrated sensor data. The results were compared, and it can be conferred that TEER and ROS sensors can serve as a substitute for conventional end-point assays.

## Experimental

### Microfluidic chip fabrication

**Fig. 1:**
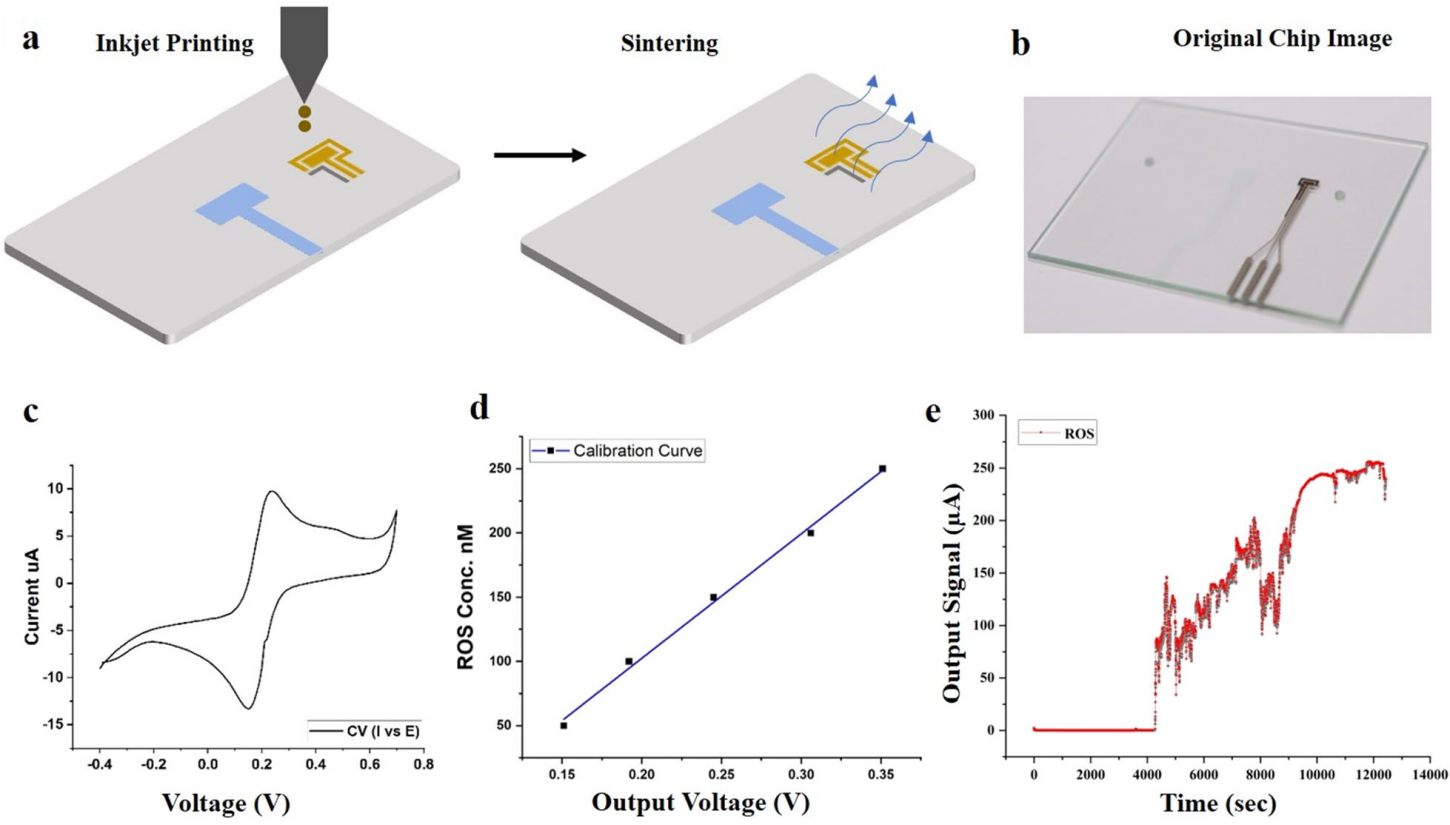
ROS sensor fabrication, characterization, and results. **a** Inkjet printing of ROS sensor pattern and sintering process **b** The real image of the printed sensor pattern, **c** CV response using potassium ferricyanide (K_4_ [Fe(CN)_6_]) and KCl. **d** calibration curve using different ROS solution at 0.65 V obtained from chrono-amperometry; **e** ROS sensor data of liver fibrosis-on-chip model with fibronectin as ECM. Real-time H_2_O_2_ concentration was monitored for 14,000 s for every second. There was no significant release of H_2_O_2_ in the fibronectin based liver fibrosis-on-chip model before the addition of the fibrosis-inducing TGF-β1 stimulation. The addition of TGF-β1 resulted in a positive output signal, and a continuous release of H_2_O_2_ was observed

The microfluidic chip was constructed with the two top and bottom glass chips (soda-lime glass, 56 mm long, 41 mm wide, and 1.1 mm thick) (Fig. 1b). A multi-head 3D printer was used to print the microfluidic channel on the top glass with PDMS (Sylgard 184, Dow Corning, USA). The glass chips were degassed thoroughly before loading into the printer stage, the fabrication height and width of the channels were set to be 300 µm and 800 µm, respectively. A customized magnetic chip holder was used to assemble the top and bottom glasses of the microfluidic chip. Additionally, the silicon gaskets were placed in the magnetic chip holder to avoid leakage.

### Cell seeding and liver fibrosis on microfluidic chip

**Fig. 2:**
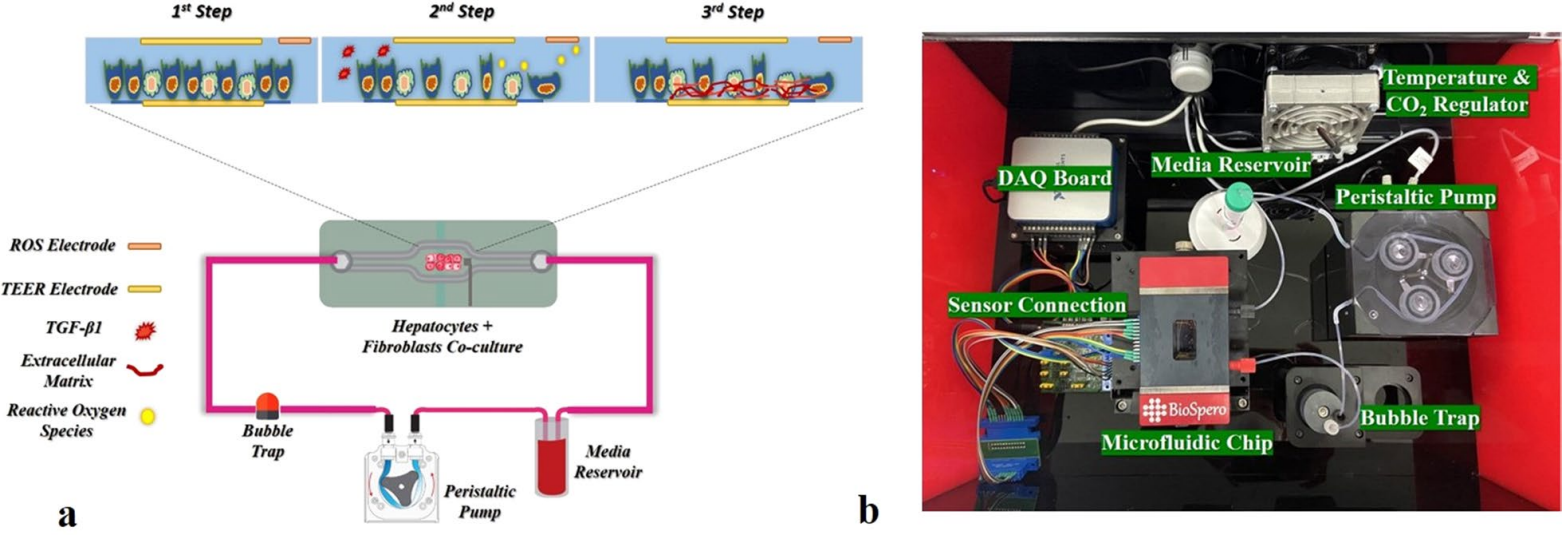
**a** The liver fibrosis-on-chip schematic; **b** The actual image of the liver fibrosis-on-chip device and associated components

A human-derived immortal HepG2 hepatoma cell line (Korea Cell Line Bank, South Korea) and human foreskin fibroblasts cell line Hs68 (Korea Cell Line Bank, South Korea) were utilized to create a co-culture model of hepatocytes and fibroblast. Both cell lines were grown in Roswell Park Memorial Institute (RPMI) 1640 cell culture media (cat# 11875093, ThermoFisher, USA) supplemented with 10% fetal bovine serum (FBS) (cat# 16000044, ThermoFisher, USA) and 1% v/v penicillin/streptomycin (P/S) antibiotic solution for cell culture (cat# 15070063, ThermoFisher, USA). The HepG2 cells and Hs68 cells were kept in a humidified incubator (with 5% CO_2_ at 37 °C). All glass chips were sterilized with 90% isopropyl alcohol and UV irradiated for 1 h in a biosafety cabinet before use. A magnetic ECM & cell seeding kit was used to apply the ECM and seeding cells on microfluidic glass chips' cell culture area. ECM solutions of collagen, poly-l-lysine, and fibronectin were used before cell seeding for cell attachment to the microfluidic chip surface. The cells were expanded and passaged thrice before seeding on the microfluidic chip at the physiological ratio of 1:8. Cells were allowed to attach the microfluidic chip for 4 h in a standard cell culture incubator at 37 °C with 5% CO_2_. After that, the glass chip top and bottom parts were assembled in a magnetic chip holder. The microfluidic chip was placed in the custom-built microfluidic platform to form a monolayer in a dynamic cell culture environment, as shown in Fig. 2b. A cell culture media reservoir of 5 mL capacity was placed and connected with the microfluidic tubing Fig. 2b. The peristaltic pump's speed was fixed at 60 μL/min to induce the shear stress of 0.5 dyn/cm^2^. Recombinant human TGF-β1 protein (cat# ab50036 abcam, USA) at the concentration of 5 ng/mL was used to induce in vitro fibrosis. The shear stress calculation formula is given below as Eq. 1. [35]

1 $$\tau = \frac{6\mu Q}{{wh^{2} }},$$

whereas here, “*μ*” signifies viscosity of the cell culture medium, “*Q*” exhibits the flow rate of the media, “*w*” represents the width and height of the channel are characterized by “*h*.”

### Sensor fabrication and characterization

TEER sensor was fabricated on both glass chips by printing a 500 nm ITO pattern using the CVD technique. The sensor was characterized as described previously [34]. Whereas the ROS sensor pattern was printed on the top glass chip downstream using an in-house multi-head 3D printer. A schematic of the process has been shown in Fig. 1a. The fabrication process involved first cleaning the substrate using ethanol, acetone, and DI water. After drying the chip surface, oxygen plasma was treated for 20 s to clean the substrate. The print speed was set at 1 ms^−1^ to fabricate gold and silver electrodes. Gold (Au) ink [cat# Au-LT-20 (20 wt%) Fraunhofer, Germany] was printed first dried at 40 °C for 10 min and sintered at 190 °C for 12 h, followed by printing the silver (Ag) ink [cat# sliver (TEC-PA-060) Solvent (DA-030) INKtec, Republic of KOREA] and sintering at 130 °C for 20 min. The sensors were characterized using a commercial PalmSens4 portable system (PalmSens, Netherlands) for cyclic voltammetry (CV) with standard solutions of 10 mM potassium ferricyanide (K_4_ [Fe (CN)_6_]) and 0.1 M KCl, as shown in Fig. 1c, d. However, a custom-developed system was used for the sensor's chronoamperometric response to plot the sensor's calibration curve, which has been shown in Fig. 2b. The sensor was then washed with Phosphate buffered saline (PBS) and double distilled water for further use in the experiments.

### ECM evaluation in the liver-on-chip device

Three different ECM have been used for studying the effect of ECM on the cell attachment to the microfluidic glass chip surface. Rat Tail Collagen (type I) (cat#C3867-1 VL, Sigma-Aldrich, South Korea) was used at concentrations of 200 µg/mL in PBS (cat# 10010023, ThermoFisher Scientific, USA). Poly-l-lysine 1 mg/mL (cat# 0403, ScienCell, USA) was diluted in sterile double distilled water to get 5 µg/mL concentrations. According to the manufacturer's instruction, fibronectin 1 mg/mL (cat# 33010018, ThermoFisher, USA) was resuspended in 1 mL of sterile double distilled water. Fibronectin was further mixed with the PBS to get the concentrations of 25 µg/mL. The 400 µL of each ECM solution was applied to the microfluidic chips' cell culture area using the ECM & cell seeding kit. The chips were then incubated overnight at 4 °C in a sterile environment.

### Albumin, urea, CYP450 enzyme measurements

Albumin, urea, and CYP450 enzyme assays were performed as functional biomarkers of the hepatocytes. Human Albumin ELISA Kit (cat# ab108787, Abcam, USA), Urea Assay Kit (cat# KA1652, Abnova, USA), and P450-Glo CYP3A4 Assay Kit (cat# V9001, Promega, USA) were used for albumin, urea, and CYP3A4 quantification, respectively. In brief, cell culture media samples were collected at specified time points and stored at − 80 °C. While CYP3A4 assay was performed by following the manufacturer’s instructions with slight modifications. Media samples were thawed at 37 °C in a water bath before experiments. A microplate reader (SpectraMax i3 Multimode Microplate Reader, Molecular Devices, USA) was used for taking readings by following the manufacturer’s instructions.

### Live/dead assay

The assay was performed according to the manufacturer’s manual (LIVE/DEAD Viability/Cytotoxicity Kit, for mammalian cells, cat# L3224, ThermoFisher, USA). Briefly, the microfluidic chip's cell culture area was washed three times with Dulbecco’s phosphate buffered saline (DPBS) and covered with a 300 µL solution of live/dead assay reagents. The chip was incubated in a humidified cell culture incubator (5% CO_2_ at 37 °C) for 30 min. Then, the cell surface was cleaned with DPBS and mounted with a coverslip. After that confocal laser scanning microscope (CLSM) (Olympus, model # FV122, Olympus, Japan) was used at the excitation of 530–560 nm and emission of 530–645 nm for taking images. The confocal images were processed for live and dead cell count by using ImageJ software (Version 1.52p, NIH, USA).

### ZO-1, α-SMA, and collagen immunofluorescence microscopy

The chip's cell culture area was rinsed thrice with pre-warmed 1× DPBS (cat# 14190144, ThermoFisher, US) solution and fixed in 4% paraformaldehyde. After that, a blocking solution (5% BSA/PBS) was used, and chips were incubated with primary antibodies against ZO-1 (cat# 33-9100, ThermoFisher, USA), alpha-smooth muscle actin (α-SMA) (cat# 14-9760-82, ThermoFisher, USA) and collagen type I (cat# PA1-26204, ThermoFisher, USA). The secondary antibodies used were F(ab′)2-goat anti-rabbit IgG (H+L) (cat# A-21430), superclonoal™ recombinant secondary antibody (cat# A28175, ThermoFisher, USA) and goat anti-mouse (H+L), super clonal recombinant secondary antibody, Alexa flour 488 (cat# A28175, ThermoFisher, USA). While 4′,6-diamidino-2-phenylindole (DAPI) (cat# 127M4055V, Sigma-Aldrich, USA) was used for cell nucleus staining. A CLSM (Olympus, model # FV122, Olympus, Japan) was used for cell visualization and capturing the images.

## Results and discussion

### Real-time monitoring of liver fibrosis-on-chip with integrated sensors

**Fig. 3:**
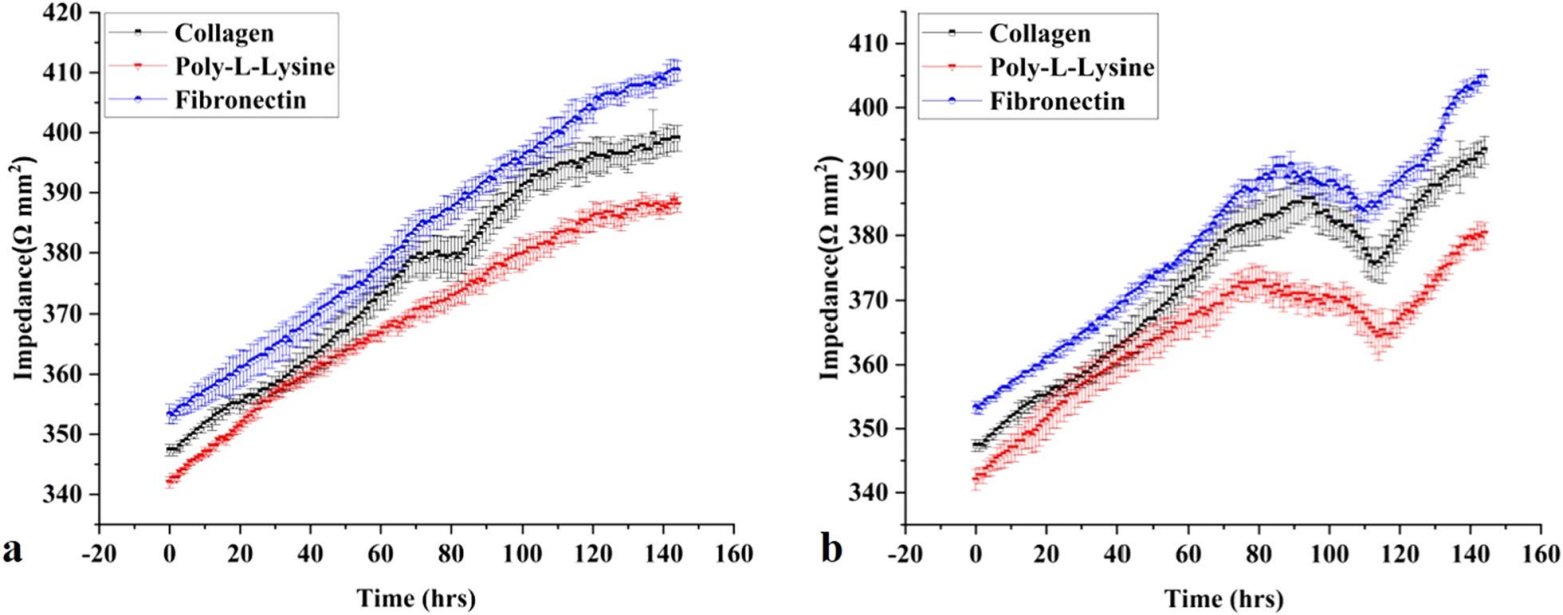
**a** Real-time TEER data graph presents the comparative impedance to different ECM time graphs in the liver MPS (data presented as mean ± SD). **b** Real-time TEER data graph illustrates the comparative impedance to the time graph of different ECM and TGF-β1 responses in the liver fibrosis-on-chip model. The TEER values increased till the formation of a monolayer at 72 h. TGF-β1 was introduced in the MPS at 72 h, which results in the drop of TEER values due to cell–cell tight junction disruption and activation of fibroblasts. Activated fibroblasts produced ECM, which eventually increased the TEER values (data presented as mean ± SD)

The microfluidic chips and platform were set up, as mentioned in Fig. 2, and the real-time monitoring of the liver MPS was carried out for 144 h for collecting control data. Static cell culture systems are facile, yet they lack mechanical stimulation such as shear stress. Physiological shear stress induces better monolayer formation and CYP450 enzyme activity [36]. Hence, the shear stress of 0.5 dyn/cm^2^ was applied to achieve better monolayer formation than static cell culture [13, 36, 37]. The TEER values of liver MPS were taken at every 1-h interval for 144 h, as shown in Fig. 3a. The TEER increase concerning the time indicated resultant co-cultured cell monolayer formation of the microphysiological environment due to the cell growth, division, and cell–cell tight junction formation. The monolayer formed on the 3rd day or 72 h. In the current study, the TEER value 370–390 Ω/mm^2^ represents a monolayer formation. However, in previous research, the TEER range of 345–395 Ω/mm^2^ indicates a compact HepG2 monolayer formation [13]. The difference from the previously reported TEER range could be due to the co-culture of HepG2 cells with fibroblasts in the present work. However, the TEER values were kept increasing throughout the experiment (Fig. 3a). It was due to the ample supply of FBS within the microphysiological system by cell culture media. 10% FBS in cell culture media results in continuously increasing TEER values and tighter cell–cell tight junction formation compared to serum-free or serum reduced cell culture media [38]. TGF-β1 in the concentration of 5 ng/mL was used as a stimulant to induce fibrosis in the liver fibrosis-on-chip model [39]. The stimulus was introduced in the chip at 72 h of the cell culture. In the liver fibrosis-on-chip model, after the addition of stimulants in the MPS, the TEER values started decreasing within 24 h (Fig. 3b). They kept dropping for 48 h due to the disruption of cell–cell TJPs. The TEER value decreased till the 105th ± 10 h, as shown in Fig. 3b. TGF-β1 is known to disrupt cell–cell TJPs in epithelial tissues [40, 41]. After that, an increase was observed in the TEER values due to the release and deposition of ECM proteins by the activated fibroblasts in the liver fibrosis-on-chip model. According to published literature, an increase in ECM deposition results in higher TEER values [42–45]. The embedded ROS sensor depicted the freedom of release of H_2_O_2_ in fibronectin based liver fibrosis-on-chip model in response to the stimulant Fig. 1e. TGF-β1 is known to induce redox imbalance in hepatocytes and produce ROS significantly, further generating a positive feedback system for activating adjacent fibroblasts [46, 47]. ROS sensor response was recorded 1 h before introducing the fibrosis-inducing stimulus (TGF-β1). There was no significant H_2_O_2_ release observed during that time. However, TGF-β1 stimulation gave rise to the production of H_2_O_2,_ and the ROS production started increasing. It is due to the negative impact of the TGF-β1 on the hepatocytes. The decrease in ROS release at the 2nd hour could be due to the reduction of cell viability [48]. Dynamic cell culture microenvironment of liver fibrosis-on-chip model results in repeated TGF-β1 exposure to resident cells. This induces more ROS production and H_2_O_2_ release with the passage of time. Overall, the ROS concentration was kept growing within the liver fibrosis-on-chip model, which can be the reason of HepG2 cell stress and fibroblasts activation and resultant ECM production.

### Albumin, urea, and CYP450 measurements

**Fig. 4:**
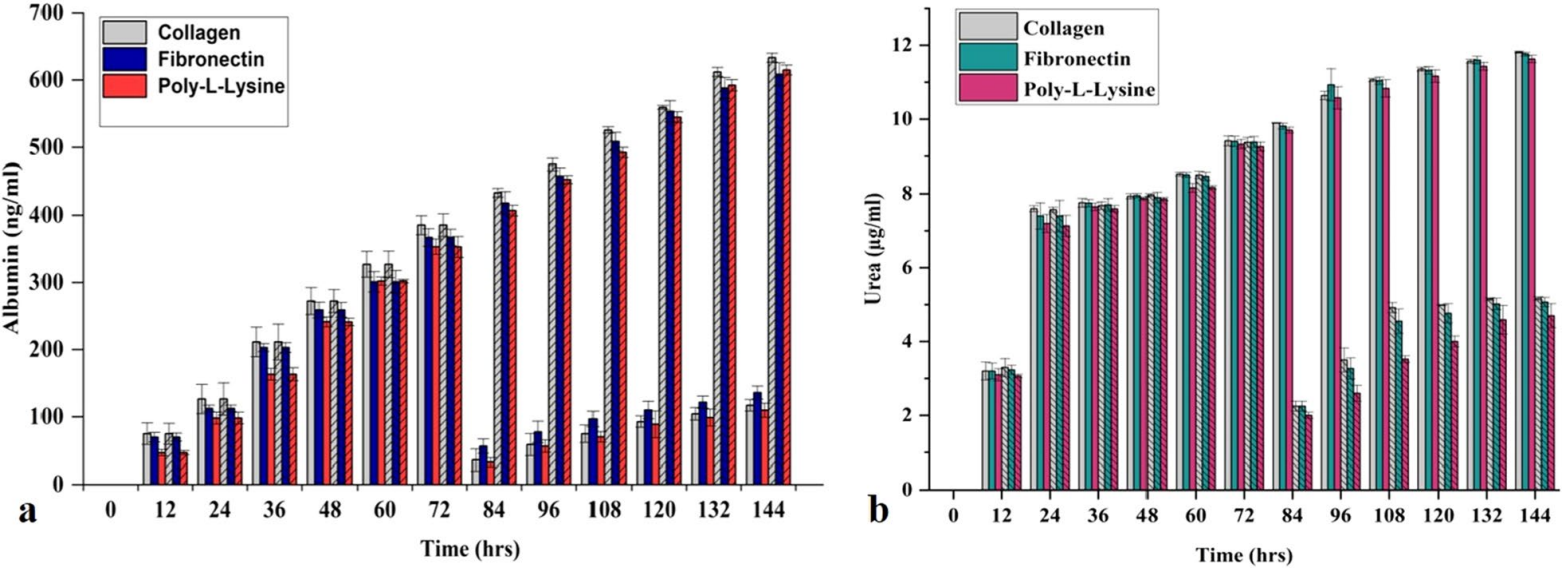
**a** Albumin release by liver MPS in control and fibrotic condition for 144 h. A sudden decrease in albumin concentration was noted after applying a fibrosis stimulus (TGF-β1) at 72 h (data presented as mean ± SD, bar column with cross lines representing the control values). **b** Urea production by liver MPS in control and fibrotic condition for 144 h. TGF-β1 stimulation for fibrosis induction at 72 h reduced the urea release by the hepatocytes [data presented as mean ± standard deviation (SD) bar column without cross lines representing the control values]

**Fig. 5:**
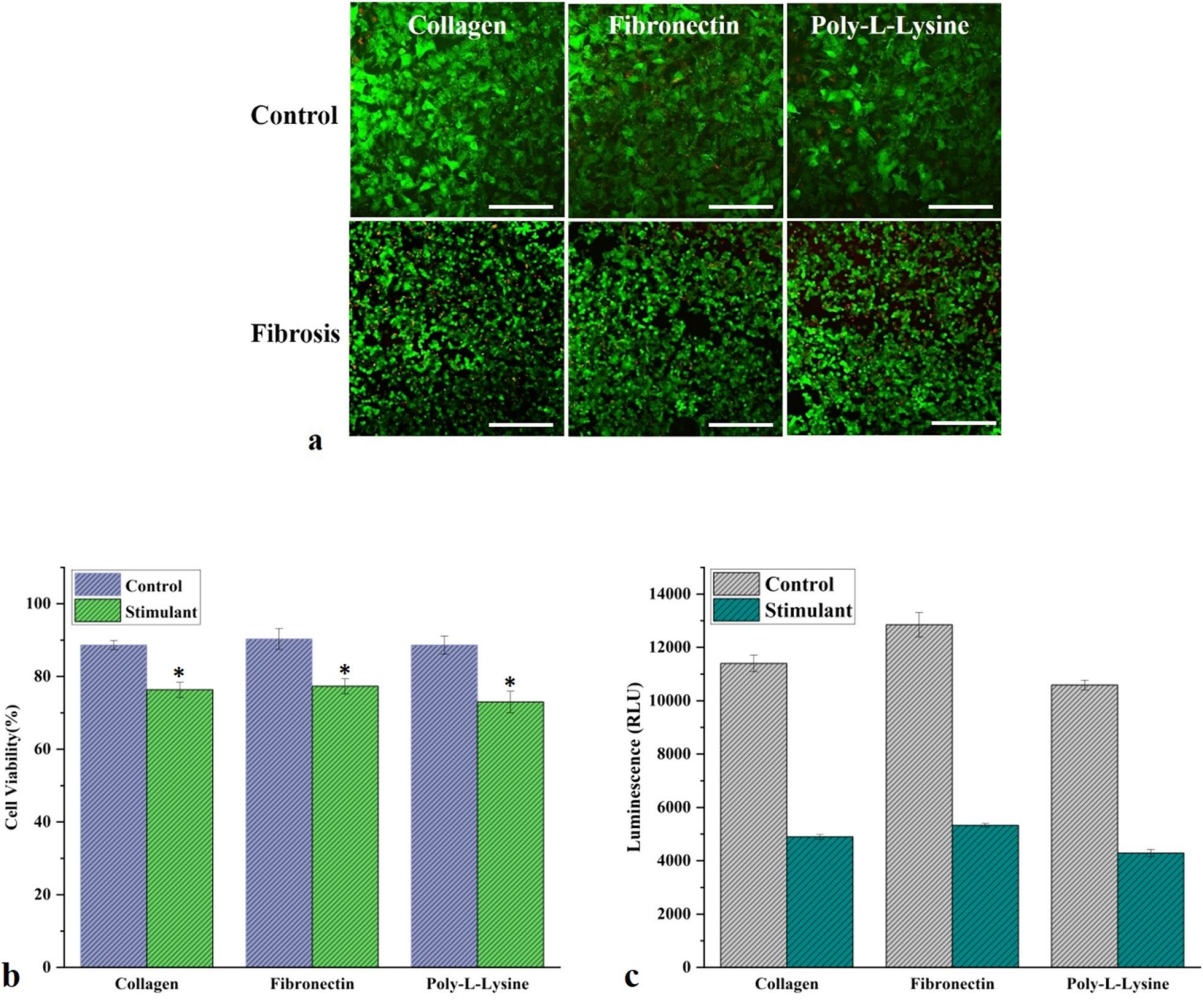
**a** The live/dead assay confocal microscopic images of liver MPS and liver fibrosis-on-chip model with different ECM. The green color is representative of live cells, while the red color is showing the dead cells. These confocal microscopy images of the live/dead assay were further analyzed with ImageJ (image processing software), and the results were displayed as a percentage (scale bar is 200 µm). **b** The live/dead assay graph shows the cell viability of control and liver fibrosis-on-chip model with different ECM. Fibronectin based liver MPS showed 90% cell viability, while 88% cell viability was observed for collagen and poly-l-lysine based liver MPS. In the liver fibrosis-on-chip model, the cell viability reduced to 77%, 76%, and 73% for fibronectin, collagen, and poly-l-lysine (data presented as mean ± SD, *represent statistical significance from the control, while the *p* < 0.05). **c** CYP3A4 expression in control MPS and liver fibrosis-on-chip model with different ECM (data presented as mean ± SD)

Albumin and urea are the functional biomarkers and reflect the pathophysiological state of hepatocytes [49]. Albumin and urea concentrations of the control MPS were measured after every 12 h until the experiment's termination on the 6th day. There was a consistent increase in the hepatocytes' albumin release, representing the MPS' overall health condition, as shown in Fig. 4a. The cell viability (live/dead assay) data further confirms the claim (Fig. 5a, b). Similarly, urea release was also consistent and physiological throughout the experiment, which depicts the MPS's physiological, metabolic condition, as shown in Fig. 1b. Cytochrome P450 (CYP450) is a group of heme oxygenase enzymes and plays a pivotal role in the metabolism of drugs and biotransformation of several biological molecules. CYP3A4 is a subgroup of the CYP450 family, responsible for 50% commercially available drugs and known to generate a significant amount of ROS during drug metabolism [50]. In liver MPS CYP3A4 activity was one fold more than static cell culture systems, which can be attributed to the shear stress and superior in vitro microenvironment compared to the conventional cell culture system (Fig. 5c) [51].

In the liver fibrosis-on-chip model, albumin and urea release compromised significantly after treatment with TGF-β1 for modeling fibrosis. TGF-β1 suppresses the activity of CYP3A4 activity and results in a lack of drug response for anti-fibrosis therapy [52]. ROS are also an essential player in reducing the efficiency of CYP3A4 by altering the protein secretion involved in autocrine and paracrine signaling [53]. ROS is often a common target of various anti-fibrosis drugs to refrain it in liver cirrhosis progression [54, 55]. During liver fibrosis-on-chip model experiments, there was a twofold decrease in the amount of CYP3A4 after TGF-β1 stimulation, as shown in Fig. 5c. TGF-β1 is known to decrease the CYP3A4 activity by several pathways, such as the hPXR pathway [52]. The addition of fibrosis-inducing stimulus also downregulated the albumin release by four-folds, as shown in Fig. 4a. In static cell culture conditions, TGF-β1 can reduce the albumin synthesis up to five folds [56]. Similarly, the urea production was also reduced in response to the stimulus Fig. 4b. It is evident from other OOC studies that stress stimuli harm hepatocyte metabolism and results in less urea formation [57–59].

### ZO-1, α-SMA, and collagen immunofluorescence microscopy and live/dead assay

**Fig. 6:**
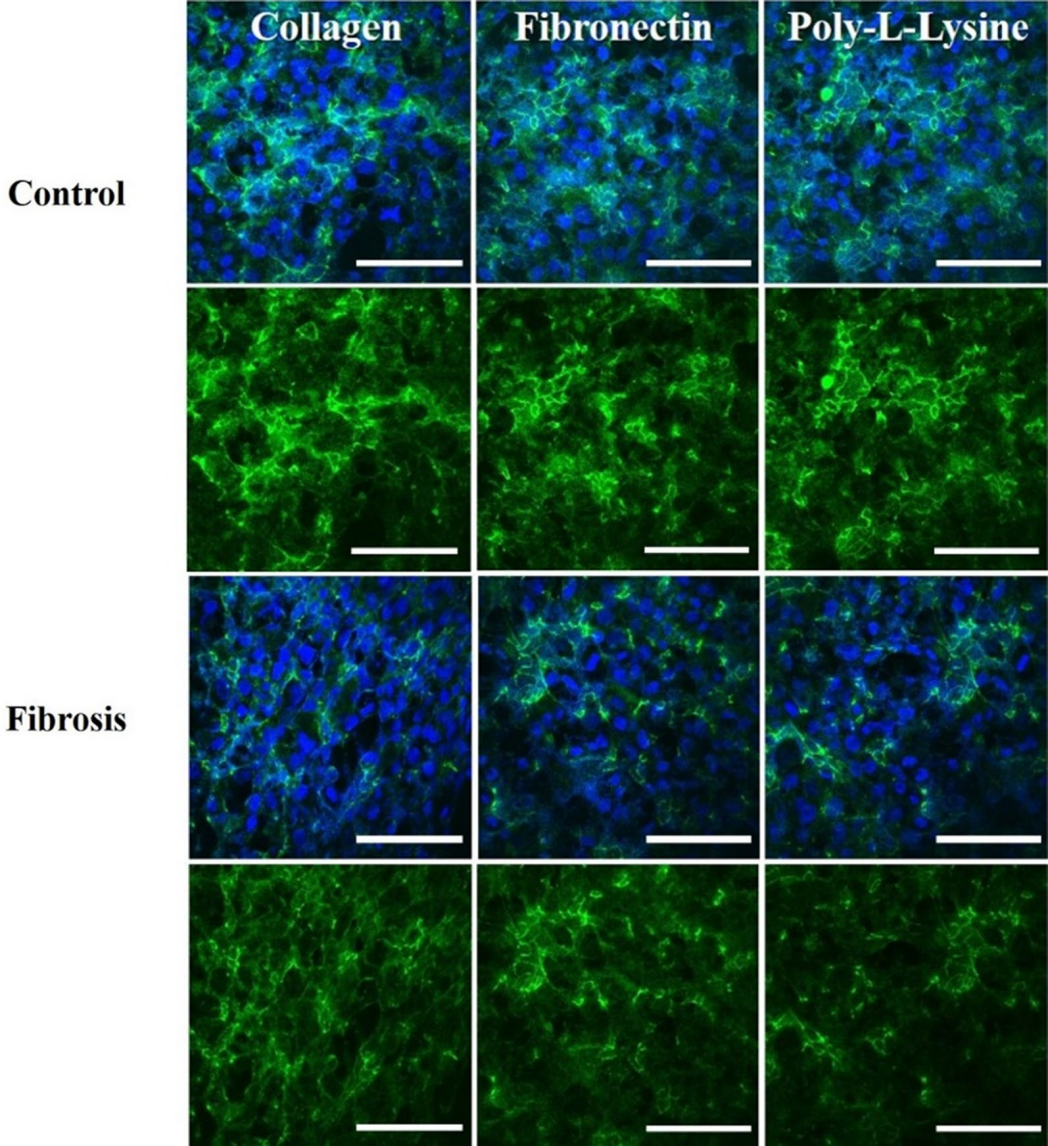
ZO-1 expression in Liver MPS and liver fibrosis-on-chip with different ECM. The control Liver MPS (without TGF-β1 stimulation) shows better ZO-1 (green color) (tight junction protein) formation. While in the liver fibrosis-on-chip model, the ZO-1 distorted. The images were taken at the termination of the experiments at 144 h (scale bar is 200 µm)

**Fig. 7:**
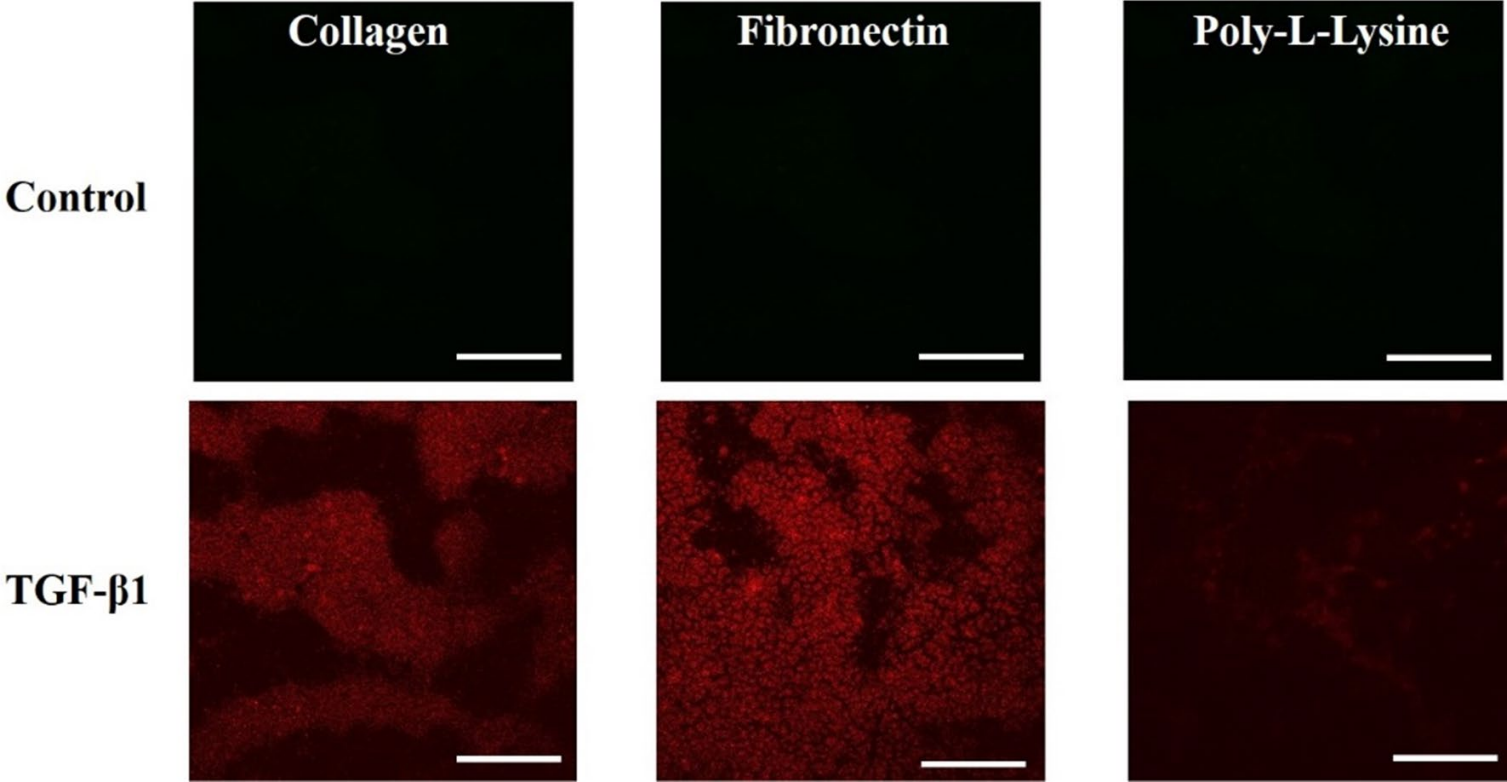
The expression of α-SMA in fibroblasts in a dynamic cell culture environment without TGF-β1 stimulation (control) and with TGF-β1 stimulation. The images were captured at 144 h (scale bar is 200 µm)

**Fig. 8:**
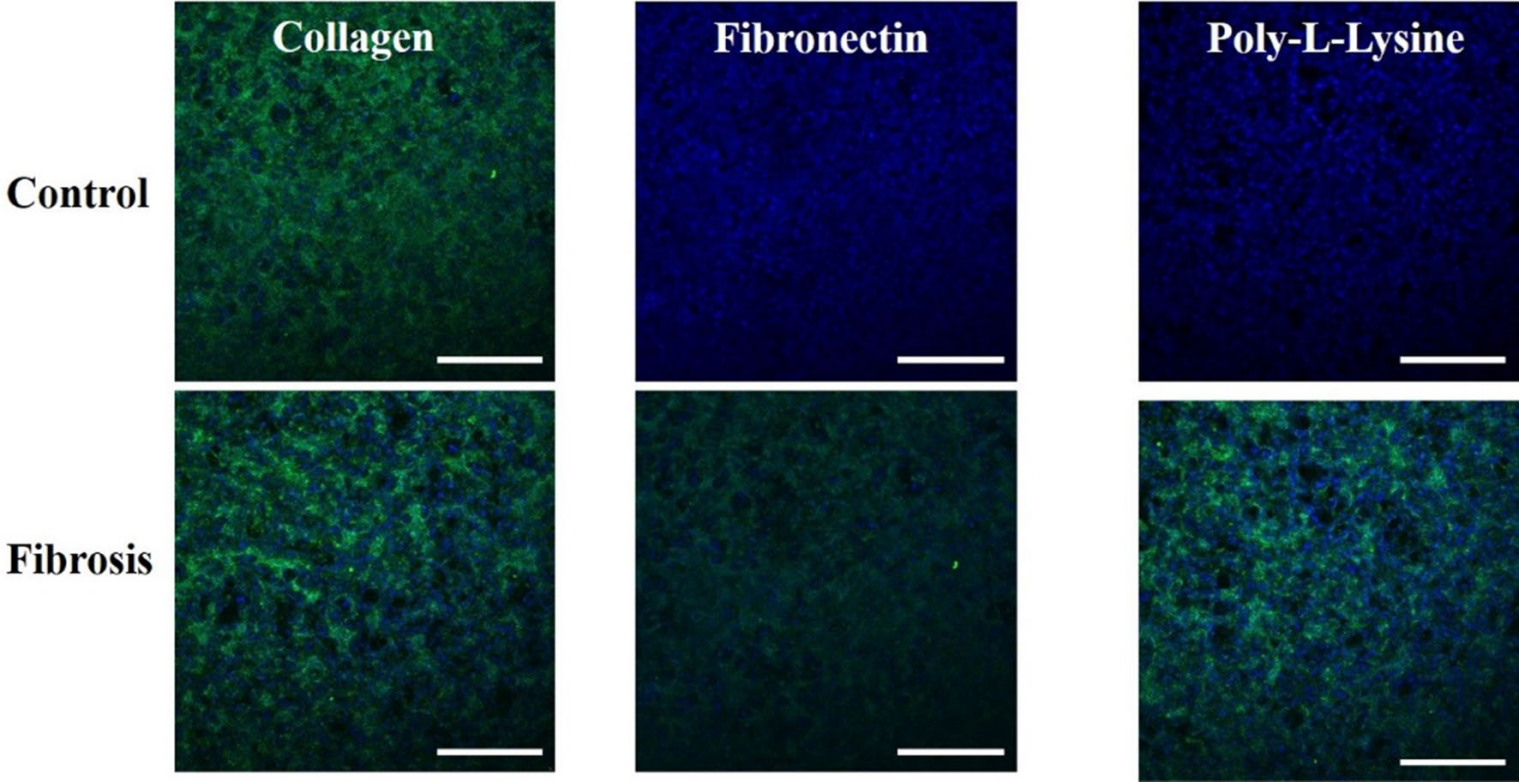
Type I collagen immunofluorescent staining images of liver MPS and liver fibrosis-on-chip with different ECM. The images are showing the expression and distribution of collagen type I (green color). The images were taken at the end of the experiments at 144 h (scale bar is 200 µm)

TJPs maintain a delicate balance to ensure a dynamic microenvironment of hepatic parenchyma and mesenchymal tissues. Pathophysiological stresses trigger the change and variation in liver TJPs. Liver TJPs are meant to change their expression in response to drugs, infections, and biological molecules. TJP-1 or zonula occludens-1 (ZO-1) protein expression was studied with the help of immunofluorescent microscopy. In control liver MPS experiments, the ZO-1 proteins were found intact around the hepatocytes. They showed tight junction formation, as shown in Fig. 6. That was the reason for the progressive increase in TEER values. α-SMA is the characteristic marker of fibroblasts activation in response to various cytokines and inflammatory mediators. Hence, fibroblasts were examined without the activation by the fibrosis-inducing factor TGF-β1. There was a negative α-SMA expression in fibroblasts (Fig. 7). TGF-β1 is known for the dissolution of TJPs through RhoA/ROCK signaling pathway in epithelial cells [60]. In the present study, the disruption of ZO-1 protein was confirmed in response to TGF-β1 stimulation, as shown in Fig. 6, which was why TEER values drop after treatment with fibrosis-inducing stimulus. In fibroblasts, treatment with TGF-β1 resulted in their activation and subsequent α-SMA expression, as shown in Fig. 7. Collagen is one of the primary components of liver ECM, and it can increase up to 10-folds during liver fibrosis [61]. Collagen immunofluorescent staining showed a significant amount of collagen deposition in the liver fibrosis-on-chip model in response to TGF-β1 stimulation compared to liver MPS, shown in Fig. 8. Liver fibrosis significantly compromises the activity and survival of hepatocytes. The deposition of increased ECM in the para and intracellular spaces disrupts the blood access to the hepatocyte and leads to cell starvation [1, 2]. The accumulation of metabolites and oxidative stress further worse the fibrotic tissue [3]. In Liver MPS experiments, the cell viability was satisfactory, as shown in the figure. While in liver fibrosis-on-chip, the cell viability decreased to 77% for fibronectin, 76% for collagen, and 73% for poly-l-lysine, as shown in Fig. 5a, b. TGF-β1 exposure resulted in compromised cellular growth and survival.

### Impact of different ECM on liver fibrosis-on-chip model

ECM is a vital component of a cell culture system and provides a niche for cell differentiation and cell division. In organ-on-chip, several ECM components such as collagen, fibronectin, fibrinogen, laminin have been used to attach the cells to a surface or a porous membrane. ECM composition and constituents vary from organ to organ [62]. However, there is no standardization or consensus among the organ-on-chip researchers about ECM's choice for modeling MPSs. Collagen is the most commonly used ECM in OOC studies. Yet, the use of poly-l-lysine in organ-on-chip technology for cell attachment is still not studied. In liver-on-chip studies, collagen and fibronectin are the most commonly used ECM [13, 59, 63, 64]. Here, we explored the choice of the most vibrant ECM for liver fibrosis-on-chip development and its effect on the TEER and ROS sensor response and biomarker yield. In control liver MPS experiments, all the ECM (collagen, fibronectin, and poly-l-lysine) provided good biomarker production and cell viability. Collagen and fibronectin were superior compared to the poly-l-lysine in terms of biomarker production and better cell viability. While fibronectin was found the most suitable ECM to model liver fibrosis-on-chip study using embedded electrochemical sensors. It has been found that all three ECM can be used for cell attachment to a glass based MPS surface. Even so, TEER values showed a variation among the use of different ECM. Poly-l-lysine based MPS showed the minimum TEER values and compared to the fibronectin and collagen-based MPS. Furthermore, ZO-1 confocal images showed lower expression of ZO-1 of poly-l-lysine based MPS. Contrary to that, fibronectin based MPS presented the highest TEER values and more ZO-1 expression than poly-l-lysine and collagen-based MPS.

There was significant variation in TEER sensor response with different ECM in the liver fibrosis-on-chip case. Fibronectin based liver fibrosis-on-chip model showed the highest TEER values compared to the collagen and poly-l-lysine coated liver fibrosis-on-chip models. It can be attributed to the fibronectin's higher molecular weight and better cell attachment of the hepatocytes and fibroblasts as compared to other ECM based liver fibrosis-on-chip models. A chip embedded ROS sensor was applied, considering the importance of the fibronectin-based liver fibrosis-on-chip model, and the H_2_O_2_ concentration was monitored in real-time.

## Conclusion

In this work, a liver microphysiological system was developed for the real-time monitoring of fibrosis development using embedded electrochemical sensors. A microfluidic glass chip was constructed, and an elastomeric microfluidic channel was fabricated onto an ITO surface using a 3D printer. CVD and 3D printing techniques were employed to print TEER and ROS sensor patterns on the microfluidic chip, respectively. A magnetic chip holder was utilized to hold together the chip components. During control experiments, sensor and biochemical assay data were collected. In the next step, fibrosis was induced in the liver MPS through a stimulus (TGF-β1), and a liver fibrosis-on-chip model was constructed. After introducing the fibrosis-inducing stimulus, the TEER values started declining, and a positive ROS response was recorded. After 24 h of stimulus activity, the TEER values began increasing due to the activation of fibroblasts and ECM deposition within the MPS system. The effect of different ECM on the sensor and biochemical response was also evaluated. It was also found that fibronectin and collagen type I are the most useful ECM for studying fibrosis development within an MPS. However, poly-l-lysine can also be employed for such experiments. In short, chip embedded electrochemical sensors can be used to monitor fibrosis-related development within an MPS.

## Data Availability

All data generated or analyzed during this study are included in this published article.

## Abbreviations

TEER: Trans-epithelial electrical resistance
TGF-β1: Transforming growth factor β1
ROS: Reactive oxygen species
TJPs: Tight junction proteins
ECM: Extracellular Matrix
HSC: Hepatic stellate cells
OS: Oxidative stress
H_2_O_2_: Hydrogen peroxide
O_2_^**.**^^−^: Superoxide anions
HO·: Hydroxyl radicals
ER: Endoplasmic reticulum
OOC: Organ-on-chip
MPS: Microphysiological systems
LOC: Liver-on-chip
ECIS: Electrical cell-substrate impedance sensing
RTCA: Roche xCELLigence Analysis
PDMS: Polydimethylsiloxane
ITO: Indium tin oxide
CVD: Chemical vapor deposition
RPMI: Roswell Park Memorial Institute
FBS: Fetal bovine serum
P/S: Penicillin/streptomycin
CV: Cyclic voltammetry
PBS: Phosphate buffered saline
CLSM: Confocal laser scanning microscope
α-SMA: Alpha-smooth muscle actin
DPBS: Dulbecco’s phosphate buffered saline
DAPI: 4′,6-Diamidino-2-phenylindole
ZO-1: Zonula occludens-1
